# An Update on the Intracellular and Intercellular Trafficking of Carmoviruses

**DOI:** 10.3389/fpls.2017.01801

**Published:** 2017-10-18

**Authors:** José A. Navarro, Vicente Pallás

**Affiliations:** Instituto de Biología Molecular y Celular de Plantas, Consejo Superior de Investigaciones Científicas, Universitat Politècnica de València, Valencia, Spain

**Keywords:** carmovirus, intracellular movement, movement proteins, Golgi, endoplasmic reticulum, mitochondria

## Abstract

Despite harboring the smallest genomes among plant RNA viruses, carmoviruses have emerged as an ideal model system for studying essential steps of the viral cycle including intracellular and intercellular trafficking. Two small movement proteins, formerly known as double gene block proteins (DGBp1 and DGBp2), have been involved in the movement throughout the plant of some members of carmovirus genera. DGBp1 RNA-binding capability was indispensable for cell-to-cell movement indicating that viral genomes must interact with DGBp1 to be transported. Further investigation on *Melon necrotic spot virus* (MNSV) DGBp1 subcellular localization and dynamics also supported this idea as this protein showed an actin-dependent movement along microfilaments and accumulated at the cellular periphery. Regarding DGBp2, subcellular localization studies showed that MNSV and *Pelargonium flower break virus* DGBp2s were inserted into the endoplasmic reticulum (ER) membrane but only MNSV DGBp2 trafficked to plasmodesmata (PD) via the Golgi apparatus through a COPII-dependent pathway. DGBp2 function is still unknown but its localization at PD was a requisite for an efficient cell-to-cell movement. It is also known that MNSV infection can induce a dramatic reorganization of mitochondria resulting in anomalous organelles containing viral RNAs. These putative viral factories were frequently found associated with the ER near the PD leading to the possibility that MNSV movement and replication could be spatially linked. Here, we update the current knowledge of the plant endomembrane system involvement in carmovirus intra- and intercellular movement and the tentative model proposed for MNSV transport within plant cells.

## Introduction

Recent phylogenetic analysis of the amino acid sequences of the RNA dependent RNA polymerase (RdRp) of members of the genus *Carmovirus* support the redistribution of 15 out of 19 of these into three different genera, *Alphacarmovirus* [type member *Carnation mottle virus* (CarMV)], *Betacarmovirus* [type member *Turnip crinkle virus* (TCV)] and *Gammacarmovirus* [type member *Melon necrotic spot virus* (MNSV)]. The genus *Carmovirus* no longer exists as such, and therefore the remaining four former members have been currently unassigned pending appropriate classification (Adams et al., 2016). In general, carmoviruses have a narrow range of naturally occurring hosts. Alpha and betacarmoviruses cause diseases mainly in flowering ornamental plants and gammacarmoviruses occur in leguminous plants, with the exception of MNSV that is confined to species in the family *Cucurbitaceae* (Hull, 2002). All of them are mechanically transmissible by grafting and/or by contact between plants. In contrast, vector and/or seed transmission have only been reported for a few species (Martini, 1958; Teakle, 1980; Krczal et al., 1995; Herrera-Vásquez et al., 2009; Mello et al., 2010; Ohki et al., 2010).

**FIGURE 1:**
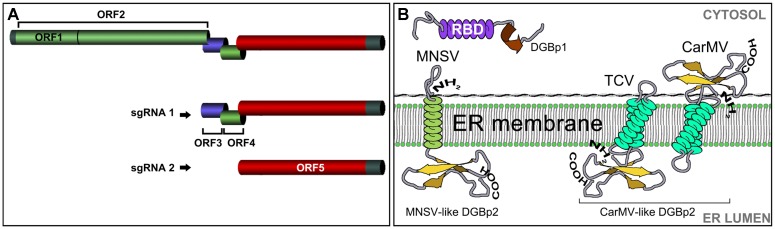
**(A)** The organization of the carmovirus genome. ORF1: auxiliary replicase; ORF2: RNA dependent RNA polymerase; ORF3: double gene protein 1 (DGBp1); ORF4: double gene protein 2 (DGBp2); ORF 5: coat protein. **(B)** The organization of the DGB genes. The first DGB ORF encodes DGBp1 having a central RNA binding domain (RBD) rich in basic residues. DGBp2 is encoded in the second DGB ORF and has one (MNSV-like) or two (CarMV-like) transmembrane domains. Topology and membrane orientation of *Melon necrotic spot virus* (MNSV), *Turnip crinkle virus* (TCV), and *Carnation mottle virus* (CarMV) DGBp2s is shown.

All viruses in these new genera have spherical virus particles of about 30–35 nm in diameter and small simple genomes comprising a single molecule of linear plus-sense single-stranded RNA, around 4 kb in length. They also share a common genomic organization consisting of at least five open reading frames (ORFs) with most of them overlapping with each other (Guilley et al., 1985; Carrington et al., 1989; Riviere and Rochon, 1990) (**Figure 1A**). The 5′-proximal ORFs are expressed from the genomic RNA while the ORFs at the 3′ half of the genome are expressed from two subgenomic RNAs (Hull, 2002). The first ORF encodes a 25–29 kDa auxiliary replicase protein and terminates in a leaky stop codon. A ribosomal readthrough mechanism generates an *in-frame* fusion product that varies from 81 to 89 kDa and corresponds to the viral RdRp. The 3-proximal gene encodes the coat protein (CP) that also acts a suppressor of RNA silencing (Thomas et al., 2003; Genovés et al., 2006; Martínez-Turino and Hernández, 2009). The CP molecular weight varies between 37 and 39 kDa with the exception of the MNSV CP that, with a size of 42 kDa, is morphologically much closer to tombusvirus than to carmovirus (Riviere and Rochon, 1990; Wada et al., 2008).

A distinctive feature in carmovirus genome organization is the presence of two centrally located ORFs encoding two small movement proteins (MPs) (6–8 and 6–13 kDa). Both have been demonstrated to be involved in cell-to-cell movement of species belonging to each new carmovirus grouping (Li et al., 1998; Genovés et al., 2006; Martínez-Turiño and Hernández, 2011). This is the reason why, by analogy with the well-known triple gene block proteins (TGBps) (Morozov and Solovyev, 2003), they have been often referred to as double gene block proteins (DGBp1 and DGBp2) (Hull, 2002).

## The Double Gene Block Proteins: Structural and Functional Features

The involvement of DGBps in cell-to-cell movement has only been demonstrated for TCV (*Betacarmovirus*), MNSV (*Gammacarmovirus*) and *Pelargonium flower break virus*, PFBV (*Alphacarmovirus*) by showing that specific mutations in either of the two DGB protein genes affected viral cell-to-cell movement (Li et al., 1998; Genovés et al., 2006; Martínez-Turiño and Hernández, 2011). The nature and the components of the carmoviruses entity that moves are not yet fully characterized but some observations suggest the CP involvement. Entire CP deletion mutants of MNSV and TCV considerably reduced the extent of local infection in melon and limited the cell-to-cell movement to a few cells in *Nicotiana benthamiana*, respectively. In both cases, the normal extent of local movement was recovered when other well-established viral suppressors were provided *in trans* (Genovés et al., 2006; Powers et al., 2008; Shi et al., 2009). Therefore, the apparent effect of these CPs on cell-to-cell movement was most likely associated with their ability to suppress RNA silencing (Qu et al., 2003; Thomas et al., 2003; Genovés et al., 2006; Serra-Soriano et al., 2017). Besides, the TCV CP was dispensable for cell-to-cell movement in *Arabidopsis thaliana* but it was essential to facilitate the entry of virions into the vasculature and cause systemic infection (Cohen et al., 2000a). Although more investigation is needed, this phenomenon could be attributable to differences in the silencing pathways between both plants. In any case, it is obvious that the intra- and intercellular transport of these viruses can take place in a non-encapsidated form in such a way that the contribution of both DGBps must be extremely relevant (Hacker et al., 1992; Cohen et al., 2000a; Genovés et al., 2006).

### DGBp1: The Putative Tractor of the Viral Genome

Secondary structure predictions and alignment of DGBp1 amino acid sequences revealed that the highest degree of similarity among them was restricted to secondary structure elements, an indicative of functional significance (Navarro et al., 2006). Studies performed with CarMV and MNSV DGBp1s, revealed the presence of an α-helical central region enriched in basic residues that modulate DGBp1 *in vitro* RNA binding (**Figure 1B**). The DGBp1-RNA recognition appears to occur via an “adaptive binding” mechanism by which both molecules change their conformation upon binding (Marcos et al., 1999; Vilar et al., 2001, 2005; Navarro et al., 2006). The DGBp1 C-terminus contains a potential β-sheet folding that can be involved in DGBp1 self-interaction (Genovés et al., 2009) and protein-protein interaction.

**FIGURE 2:**
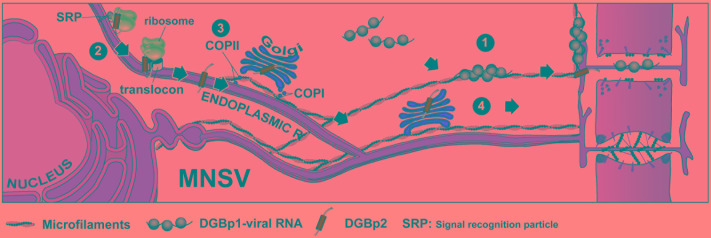
Intracellular transport of double gene block proteins of *Melon necrotic spot virus*. MNSV DGBp1 and DGBp2 likely use two independent pathways to reach the cellular periphery and PD. Self-interacting DGBp1 molecules may bind vRNA to form RNPs that associate with actin microfilaments and move to cellular periphery in an actin-dependent manner (1). After cotranslational/signal recognition particle (SRP)-dependent and translocon-assisted insertion into ER membranes (2), MNSV DGBp2 is exported to the Golgi apparatus in a COPII-dependent pathway (3). Next, DGBp2 is targeted to PD via an unidentified post-Golgi route, or alternatively it can reach the PD by lateral diffusion along the ER (4).

The ability of the strain B of TCV to cause larger lesions and systemic symptoms in *A. thaliana* in a higher percentage than that caused by the strain M was related to a difference in the affinity for RNA between their corresponding DGBp1s, and it was specifically due to a change from lysine in TCV-M DGBp1 to glutamate in TCV-B DGBp1 (Wobbe et al., 1998). MNSV cell-to-cell movement was extremely intolerant to any amino acid change that either reduced the global charge or provoked the destabilization of the secondary structure of the DGBp1 central region. Nevertheless, the Nt and Ct regions of the MNSV DGBp1 allowed a reduction in the number of basic residues before the movement was completely abolished. In these latter cases, the extent of the local infected area of each MNSV mutant directly correlated with the RNA affinity of the corresponding DGBp1 mutant protein. In view of these findings, DGBp1 stands as a strong candidate for being the factor that binds the viral genome in order to initiate the transport toward the plasmodesmata (PD). It is well-known that plant viruses need to exploit PD to move from cell to cell and, in this sense, ectopically expressed MNSV DGBp1 was localized in motile granules that associated to actin microfilaments (MFs), showed actin-dependent movement at the cytoplasm and finally accumulated at the cellular periphery near the PD (**Figure 2**). These DGBp1 mobile granules were not related with the major membrane bounded organelles and therefore could represent inclusion bodies consisting of self-interacting molecules (Genovés et al., 2009). Furthermore, subcellular fractionation experiments showed that, later in infection, CarMV DGBp1 mainly associated to the cell wall fraction (Garcia-Castillo et al., 2003). In contrast, TCV DGBp1 showed a nuclear localization mediated by two nuclear localization signals (NLSs) that were necessary for cell-to-cell movement (Cohen et al., 2000b).

### DGBp2: The Membrane Associated Component

The second MP found in carmoviruses (DGBp2) has been classified into two subgroups, namely MNSV-like and CarMV-like, depending on whether it has one or two potential transmembrane domains (TMDs) (Navarro et al., 2006; Serra-Soriano et al., 2014). CarMV-like DGBp2s such as those of CarMV and TCV have been reported to be targeted and inserted *in vitro* into canine pancreatic rough ER-derived microsomes by means of a cotranslational/signal recognition particle (SRP)-dependent and translocon-assisted process (Sauri et al., 2005; Navarro et al., 2006; Martínez-Gil et al., 2007, 2010). Their membrane topology was determined *in vitro* and the N- and C-terminal regions of the CarMV DGBp2 faced the cytoplasm whereas TCV DGBp2 showed an opposite orientation with both termini exposed to the ER lumen (**Figure 1B**) (Vilar et al., 2002; Martínez-Gil et al., 2007).

The MNSV and PFBV DGBp2s have also been demonstrated to associate to plant ER membranes but only the former was shown to be further targeted to PD via the Golgi apparatus (GA) in a COPII-dependent pathway (**Figure 2**) (Genovés et al., 2011). The PFBV DGBp2 differs from other CarMV-like DGBp2s in having an N-terminal extension with RNA-binding capacity and a leucine zipper-like motif that is probably involved in protein-protein interactions. PFBV DGBp2 ER export to other cellular membranes cannot be excluded since it was occasionally observed in unknown punctuate structures at the cytoplasm and close to the plasma membrane (Martínez-Turiño and Hernández, 2011). Genovés et al. (2011) also showed that the MNSV DGBp2 has a type II topology (Ncyt-Clum, **Figure 1B**) in plant cells as was previously described using bacteria (Martínez-Gil et al., 2007), but topological rules applicable in each case were different. MNSV DGBp2 mutational analysis revealed that neither a twofold increase in TMD hydrophobicity (von Heijne, 2007) nor mutations that violate the positive-inside rule (Lerch-Bader et al., 2008) resulted in opposite membrane orientation. Moreover, distortion of the TMD secondary structure was the unique determinant affecting the MP-membrane association suggesting a robust interaction with the ER. Despite this, most mutant proteins displayed an incorrect intracellular targeting to unidentified cytoplasmic vesicles or instead they produced a drastic rearrangement of ER membranes that impeded the protein to access the PD. After introducing these mutations into MNSV infectious RNAs, it was observed that the DGBp2 ER-to-GA transport was essential but not sufficient to promote cell-to-cell movement that strongly required the PD localization of the MP (Genovés et al., 2011). Although the plant GA has been reported to be the default destination for single-pass membrane proteins containing TMDs of about 19–20 amino acid in length (Brandizzi et al., 2002), the TMD of the MNSV DGBp2 by itself was unable to promote the ER-to-GA transport of the green fluorescent protein (GFP), resulting in ER retention. Actually, MNSV DGBp2 ER export was dependent on the specific contributions from a cytosolically exposed β-turn comprising DSSP residues and a luminal-oriented lysine (K49). Cytosolic exposure of the DSSP motif was essential to trigger Sar1 recruitment to ER membranes and protein ER export. Sar1 is a small GTPase that promotes the coat protein complex II (COPII) assembling and the formation of ER-to-GA transport vesicles. Mutations in the DSSP motif barely allowed MNSV to move into a few adjacent cells. These DGBp2 mutant proteins accumulated at the cellular periphery when it was transiently expressed suggesting that they still can reach the PD by lateral diffusion along the ER membranes. Therefore, DGBp2 needs to be targeted to the PD through the secretory pathway in order to achieve an efficient and fast viral transport to adjacent cells. Moreover, K49 was shown to be necessary for actin-dependent lateral diffusion of DGBp2 into the ER membranes, a condition that was essential for ER export. It was suggested that the connection of DGBp2 to the actin-ER network must require the interaction with an actin-associated transmembrane adaptor (Serra-Soriano et al., 2014). In this sense, it was shown that removing the anchoring aromatic amino acids that flank the MNSV DGBp2 TMD allowed the mutant virus to move farther than the wild type (Genovés et al., 2011).

Although the specific role of DGBp2 in viral transport is as yet unknown, an early model was proposed in which the DGBp2 cytosolic faced regions would interact with DGBp1 bound in turn to the viral RNA. This ternary complex would be translocated from the sites of replication to PD through the endomembrane system in a DGBp2-driven process (Vilar et al., 2002; Navarro et al., 2006). This was a striking and simple model, but it should be emphasized that MNSV DGBp1 and DGBp2 move intracellularly toward PD in the absence of other viral products through different and independent routes (**Figure 2**). These findings do not exclude the possibility that interaction between both DGBps takes place in the cellular periphery and, especially in the PD. This model is, however, hardly extensible to all carmoviruses. For instance, none of the TCV DGBps appears to be associated with punctate structures at the cell periphery or with the cytoskeleton. In addition, TCV DGBp2 was found in both the nucleus and cytoplasm (Cohen et al., 2000a). Thus, it appears that the mechanism governing carmovirus movement could be more intricate than previously though.

## Replication and Movement Connection in Carmovirus

It is well-documented that viral RNA molecules must associate with MPs in order to be transported within and between cells. However, where and when this encounter should take place in the cell is still an open issue. Most positive-strand RNA viruses replicate their genomes in association with membrane structures derived from the ER and GA or from membrane-bound organelles (Verchot, 2011; Grangeon et al., 2012; Romero-Brey and Bartenschlager, 2014). This association leads to cytological modifications that cause a great impact within the cell by producing membranous compartments that, even morphologically different, can support viral complex assembling and replication (Jiang and Laliberté, 2016). The most remarkable cytological modification induced by carmoviruses is frequently related to mitochondria alterations. For example; PFBV induced enlargements of the mitochondrial cristae which often contained fibrillar material (Lesemann and Adam, 1994); an extensive peripheral vesiculation of mitochondria was observed in TCV-infected turnip cells (Russo and Martelli, 1982; Blake et al., 2007); and mitochondrial ultrastructural changes present in MNSV-infected melon cells were also dilated cristae, an evident matrix double membrane separation and rather large dilations inside them. Besides, dsRNAs were detected in these altered organelles (Gómez-Aix et al., 2014).

These findings make the mitochondria a plausible candidate to be the site for carmovirus replication. In fact, several reports with members of the three carmovirus genera have shown that the auxiliary replicase protein is targeted to mitochondria: immunoblot analysis of mitochondrial proteins from storage roots indicated that TCV p28 was associated with the mitochondria outer membrane (Blake et al., 2007); GFP fusions of p27 and RdRp (p86) of PFBV were located in mitochondria from plant and yeast cells (Martínez-Turino and Hernández, 2012); and fractionation of tissues expressing MNSV p29 and confocal imaging using GFP-p29 also shown that p29 associated with the mitochondrial membrane (Mochizuki et al., 2009). Based on data from related tombusviruses, it was proposed that the auxiliary replicase facilitates the formation of multimolecular complexes by interacting with the RdRp, the viral genome, and unidentified host factors. It is also assumed that the auxiliary replicase determines the subcellular localization of the replication complex (Panavas et al., 2005). One important point to consider in looking at this scenario is whether virus replication and movement are spatially separated or linked processes. A tightly association between replication and movement has been reported for a few viruses such as *Tobacco mosaic virus* (Kawakami et al., 2004; Liu and Nelson, 2013), *Red clover necrotic mosaic virus* (Kaido et al., 2009), *Potato virus* X (Tilsner et al., 2013), and *Turnip mosaic virus* (Grangeon et al., 2013). In the case of carmoviruses, however, it remains unclear how replication and movement are orchestrated.

Gómez-Aix et al. (2014) elegantly addressed the cytopathology of MNSV in melon cotyledons by transmission and focused ion beam-field emission scanning electron microscopy. They found that most of the mitochondria in infected cells contained large dilations enclosing numerous vesicles of 45–50 nm in length that were also observed along their external membrane. Interestingly, these vesicles were focusing toward the cytoplasm in close relationship with the ER. Altered mitochondria also contained viral particles and most likely unassembled CP as well as viral single and double stranded RNA molecules. In the same work, it was demonstrated that p29 could induce mitochondria reorganization and formation of structures with numerous dilations surrounded by double membranes. These findings strongly indicated that MNSV replication takes place in these abnormal organelles.

It has been shown that a protein complex, named endoplasmic reticulum (ER)-mitochondria encounter structure (ERMES), is located at the interface of the ER and the mitochondria providing a hub for fine communication between both organelles (Mueller and Reski, 2015). Although ERMES was not involved in PFBV p27 targeting to mitochondria in yeast (Martínez-Turino and Hernández, 2012), it is tempting to speculate that MNSV could take advantage of this complex to interconnect replication and movement. Interestingly, Gómez-Aix et al. (2014) reported a frequent association of the MNSV viral factories to the ER in close proximity to the PD. This observation may suggest the presence of intercellular transport mechanisms functioning more directly in a similar way to that described for *Potato virus* X where viral RNA is coreplicationally delivered into the PD (Tilsner et al., 2013).

## Conclusion and Perspectives

Despite having the smallest genomes in the viral RNA world carmoviruses have turned to be ideal model systems for the study of essential processes for the viral cycle such as translation (Gao et al., 2012; Simon and Miller, 2013; Miras et al., 2014; Blanco-Perez et al., 2016), movement (Serra-Soriano et al., 2014), RNA silencing (Zhou et al., 2008; Zhang et al., 2012), membrane insertion (Vilar et al., 2002), and resistance mechanisms (Chandra-Shekara et al., 2004; Nieto et al., 2006). Although a significant progress has been made in knowing the viral determinants underlying these essential processes, the cellular aspects that could shed light on the real scenario in which these processes take place are lacking. It is evident that there is a need to study the subcellular localization of DGBps and their interactions during viral infection and to know more about how the virus-induced subcellular structures condition the plant cell function and allow viral infection. In addition, the physical entities that cross plasmodesmata or translocate through the phloem to allow the systemic invasion of the plant are far from being elucidated. Finally, during the last years an increasing number of cellular pathways have been reported to be remodeled by plant RNA viruses. Carmoviruses can be an ideal model to study how these pathogens subvert these pathways in their own benefit and can help to understand the mechanistic insights that operate at the cellular level.

## Author Contributions

All authors listed have made a substantial, direct and intellectual contribution to the work, and approved it for publication.

## Conflict of Interest Statement

The authors declare that the research was conducted in the absence of any commercial or financial relationships that could be construed as a potential conflict of interest.
